# Recognition and management of haemophagocytic lymphohistiocytosis on the ICU: a case series

**DOI:** 10.1186/cc11048

**Published:** 2012-03-20

**Authors:** R Baumber, B Agarwal, M Carrington

**Affiliations:** 1 Royal Free Hospital, London, UK

## Introduction

Haemophagocytic lymphohistiocytosis (HLH) is a rare haematological condition, with a reported incidence of the familial variety of the disease being 1.2 cases per 10,000 children per year. The acquired form of HLH predominantly affects adults and is almost always precipitated by infection, with Epstein-Barr virus (EBV) being the commonest trigger. This results in abnormal activation and proliferation of histiocytes and macrophages. Widespread phagocytosis of blood cell components leads cytopenias, a strong proinflammatory response and cytokine release leading to tissue necrosis and multiple organ failure. A large majority of these patients will present to the ICU for organ support and the aim of this report is to provide an update on HLH and raise awareness of this rare condition amongst the critical care community.

## Methods

A single-centre retrospective review of case records of all patients admitted to our ICU, in a tertiary haematology referral centre, in the last 5 years with a confirmed or suspected diagnosis of HLH, based on HLH-2004 guidelines. Data were collected on demographic variables, HLH disease characteristics, acute physiological derangement (APACHE II and SAPS II), and outcome.

## Results

Twenty-four patients were identified with a diagnosis of HLH, 18 males and six females, with mean age 42.6 years. A history of prior haematological malignancy, HIV infection and immunosuppressive therapy was present in six, five and four patients respectively; no underlying medical condition was found in 5/24 patients. Infective causes were identified in 15/24 patients, EBV in eight out of 15. Other infective causes were Cytomegalovirus, *Toxoplasma gonadii*, *Mycobacterium tuberculosis *and Schistosomiasis. All patients were pancytopenic at ICU admission and had significantly elevated serum ferritin (15,771 ± 17,718) and triglyceride (3.8 ± 2.05) levels. Eleven out of 24 patients displayed features of acute liver involvement. Mean APACHE II score was 20.5 ± 5.1 and mean SAPS II was 51.3 ± 12.1. Ten out of 24 survived to ICU discharge, and 6/24 (25%) were alive at the time of hospital discharge. The survivors had lower APACHE and SAPS scores, and were associated with a non-EBV infection and a lower incidence of liver involvement.

## Conclusion

HLH is a rare but fatal haematological syndrome that in its acquired form may present to ICU clinicians for organ support. Diagnosis of HLH in the intensive care setting may be difficult because sepsis may cause similar clinical and laboratory abnormalities. Presence of more severe acute physiological derangement, EBV aetiology and features of liver failure portend a poor prognosis in HLH.
